# Histological Evaluation of Root Canals by Performing a New Cleaning Protocol “RUA” in Endodontic Surgery

**DOI:** 10.3390/dj11030078

**Published:** 2023-03-09

**Authors:** Alfredo Iandolo, Alessandra Amato, Massimo Pisano, Giuseppe Sangiovanni, Dina Abdellatif, Roberto Fornara, Michele Simeone

**Affiliations:** 1 Department of Medicine and Surgery, University of Salerno, 84084 Salerno, Italy; 2 Department of Endodontics, Faculty of Dentistry, University of Alexandria, Alexandria 21526, Egypt; 3 Private Practitioner, 20010 Milan, Italy; 4 Department of Endodontics, Faculty of Dentistry, University of Naples, Federico II, 80126 Naples, Italy

**Keywords:** cleaning, EDTA, endodontic surgery, ultrasonic activation

## Abstract

Aim: To enhance cleaning during retro-preparation in endodontic microsurgery. Materials and Methods: Forty mandibular premolars were instrumented, filled with a single cone technique, and then retro-preparation was performed and assigned to experiment A. In group A1, the cavity created by the retro preparation was cleansed with 2 mL of normal sterile saline. In group A2, the retro cavity was cleaned with 2 mL of sterile saline after the retro preparation. All the irrigation solutions mentioned above were delivered using an endodontic needle with a lateral vent and a gauge of 30. Subsequently, in group A2, 17% EDTA gel and 5.25% gel were inserted into the cavity and activated using ultrasonic tips. After the irrigation protocols, the specimens were decalcified for histological evaluation. Results: In the experiment, the amount of hard tissue debris was significantly greater in group A1 compared to group A2 (*p* < 0.05). Conclusions: The samples in group A2, where the new protocol was performed, showed statistically significant results.

## 1. Introduction

Endodontics is considered the dental science that deals with the internal tissues of the tooth regarding their pathologies and related treatments [1]. The goal of endodontic treatment is not only to achieve short-term success but to aim for long-term success as well. Moreover, to achieve that objective, the treatment should be enhanced by adequate 3D cleaning of the complex endodontic space after root canal shaping, followed by complete 3D obturation of the complicated endodontic system [2]. Looking deeper into the root canal space, macroscopically, it is formed of areas easily accessible to hand and rotary instruments (the main root canals), as proved by many clinical and histological studies. Moreover, examining the root canal more profoundly, some spaces are primarily hard to access or even inaccessible (isthmuses, loops, lateral canals, ramifications, deltas, and dentinal tubules) [3,4].

Furthermore, these zones have been cleaned; they can then be filled with gutta-percha and sealer during the obturation phase [5,6].

By surgical endodontics, we indicate that the branch of dentistry that deals with diagnosing and treating lesions originate from an endodontic source unresponsive to orthograde endodontic treatment. Furthermore, it can be applied in cases where it is impossible to have orthograde access [5].

Surgical treatment carried out with modern techniques is a predictable choice that, even after a year of follow-up, guarantees 91.6% of success [6]. It is most useful in cases with a periapical lesion in teeth in which an adequate orthograde treatment has been carried out or complex restoration is present [7]. 

In the decision-making phase, total aid for the endodontist is represented by the CBCT, which facilitates the planning of the surgical intervention, allowing for the evaluation of missing canals, resorptions, perforations, and complex canals with greater precision anatomies and the proximity of the anatomical structures [8]. 

In the 90s, the introduction of the operating microscope [9], combined with ultrasonic retro-tips, opened the doors to a new era of surgical endodontics (the micro-ultrasonic era).

In surgery, the operating microscope permits unparalleled precision; chiefly, it facilitates exposing the root apices with minimal damage [10]. Concurrently, the use of ultrasonic instruments leads to the removal of a lesser amount of bone, with lower resection angles (which allow preserving the cortical bone and not excessively decreasing the length of the root), as well as allowing the creation of the cavities parallel to the long axis of the root following the canal perpendicularly for a few millimetres [11]. 

Moreover, with adequate magnification and illumination, it is easier to identify anatomical variables in a sectioned root, such as isthmuses, canal confluences, micro-fractures, and lateral canals, which can lead to therapy failure [12]. 

This present work aims to introduce a new irrigation protocol, Retro Ultrasonic Activation (RUA), of the apical retro-cavity for better cleansing of the canal walls, which can further reduce the bacterial existence and ensure greater chances of clinical success, allowing, for these results, even the minimum apical resection.

The null theory experimented with was that there would be no difference in debris removal between the traditional irrigation and the proposed active technique after performing the retro-preparation.

## 2. Materials and Methods

### 2.1. Specimen Selection

The research was conducted in consonance with the guidelines of the Declaration of Helsinki and permitted by the Institutional Review Board: protocol code 55/21, 28 April 2021, Naples University, Federico II, Italy.

Extracted mandibular premolars (*n* = 40) were picked for this research based on a protocol authorised by the Institutional Review Board and Ethics Committee. These mandibular premolars were extracted as part of an orthodontic treatment plan and were irrelevant to the current work. Consent forms were acquired from all the patients prior to the treatment. 

Exclusion criteria were: fracture, crack, resorption, periapical lesion, previously treated tooth, history of trauma, immature apex, and calcification. Inclusion criteria were: normal anatomy, single root canal, and sound teeth extracted for orthodontic purposes.

The periodontal tissues on the outer root surfaces were removed. The samples were then preserved in separate containers with 5 mL of 10% formalin solution up to usage [12].

### 2.2. Root Canal Preparation

One expert operator with more than 20 years of expertise conducted the experiments.

The premolars were cropped at the CMJ (cement-enamel junction) to create identical specimens 18 mm in length. The working length was established using a 0.10 hand K-file (Coltene/Whaldedent Inc., Cuyahoga Falls, OH, USA). The file was introduced in the canal until seen from the apex, and the working length was 0.5 mm less than that measurement. The root canal shaping was performed with Ni-Ti (nickel-titanium) rotating instruments (Coltene/Whaldedent Inc., Cuyahoga Falls, OH, USA). The 10/0.05, 20/0.05, and 25/0.08 one file of Hyflex EDM was utilised to shape the root canals to the complete WL mechanically. While shaping the canals, chemical cleaning was performed with 3% sodium hypochlorite (Coltene/Whaldedent Inc., Cuyahoga Falls, OH, USA) using a 30G open-vented needle in a syringe (Coltene/Whaldedent Inc., Cuyahoga Falls, OH, USA). A total of 5 mL of NaOCl was applied to each canal and revived every 60 s. Afterwards, the canals were irrigated with normal saline and 3 mL of 17% EDTA (Coltene/Whaldedent Inc., Cuyahoga Falls, OH, USA). The latter solution was left in the canal for 60 s to clear the formed smear layer. All root canals had the last rinse of 3 mL of normal saline.

After drying the instrumented and cleaned root canals with sterile paper points, the roots were obturated. The obturation was performed using the single cone approach with the sealer Bioseal (Coltene Whaldedent Inc., Cuyahoga Falls, OH, USA) and 25/0.08 gutta-percha cones (Coltene Whaldedent Inc., Cuyahoga Falls, OH, USA). Then, the samples were kept in a saline solution for seven days [12,13].

### 2.3. Assessment of Root Canal Cleanliness

The collected premolars were allocated into groups 1 (*n* = 20) and 2 (*n* = 20). All the roots of each experiment were secured through a Morse. Afterwards, the apicoectomy was created in all the samples by reducing 1 mm of the apex of the teeth using a multi-blade safe end bur. Then, the depth of the retro preparation was conducted for 3 mm utilising the retro tip P14D (Satelec, 33700 Merignac, France). All procedures were performed at 8× magnification using an operating microscope (Kaps, Som 32, Karl Kaps GmbH & Co. KG, Schulstraße 57, 35614 Asslar/Wetzlar).

After completion of the retro preparation, the teeth were divided into two groups according to the cleaning technique used in the retro cavity:

Group 1: 2 mL of sterile saline was used through a gauge size 30 root canal needle and parched using sterile paper points.

**Figure 1:**
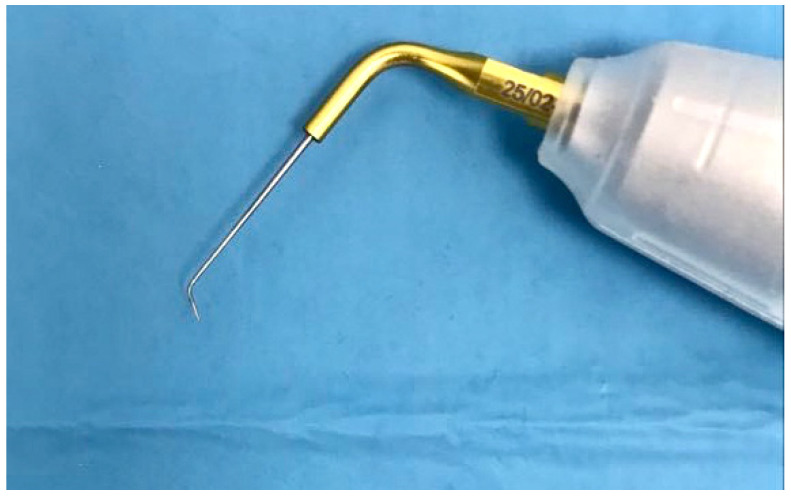
Modified ultrasonic tip.

Group 2: 2 mL of sterile saline utilising a 30-gauge endodontic needle was applied, and then, the cavities were filled with 17% EDTA gel. The latter was subjected to ultrasonic activation for 30 s using a modified ultrasonic tip (25/02, Ultra Smart AI, Coxo, China), as displayed in Figure 1. The ultrasonic tip was bent with a 100° angulation in the final 3 mm using the Endo-bender (Kerr, CA 92821 California), and the 100° angulation was adjusted according to a goniometer. EDTA was then deactivated and flushed out using 1 mL of 5.25% NaOCl gel by a 30-gauge endodontic needle. Then, another 1 mL of 5.25% NaOCl gel was introduced again and ultra-sonically activated in the same way as the EDTA. Ultimately, the root end cavity was dried using paper points [11].

Next to the irrigation protocols, the specimens were dried and fixed using a 4% buffered formalin liquid for 48 h, washed underneath running water for 1 h and then submerged in Morse solution for 28 days. The decalcification solution was replenished every 48 h. At the end of the four weeks, six-micron-thick serial cross-sections were acquired from the roots based on a formerly explained protocol. A whole of 10 serial sections at 1–3 mm from the apex of the teeth were stained with hematoxylin and eosin. After that, the slides were investigated under an optical microscope (OptiKa TB 290, Optika, Turin, Italy) at 40×, 100×, and 200×, employing the dedicated Otpika Vision Lite software.

The quantity of tissue debris present in each section was graded by two independent, blinded, calibrated operators (D.A, R.F) established on the following criteria: grade 1: the existence of debris all over the area, grade 2: the presence of debris more increased than 50% of the entire area, grade 3: the existence of debris higher than 25% of the complete area, and grade 4: absence of debris or less than 25% throughout the area (Table 1) [14].

In case of conflict between the assessors, a third assessor scored the specimens, concluding scores following a discussion between the three assessors.

### 2.4. Statistical Analysis

Non-parametric tests were used for multiple group comparisons (Kruskal–Wallis). Medians and interquartile ranges for experiment A in groups 1 and 2 were computed. The Mann–Whitney U test was employed to compare the medians between groups 1 and 2 for experiment A. A standard statistical software package (SPSS, version 27.0; SPSS IBM, Armonk, New York, NY, USA) was used. The level of significance was set at *p* < 0.05.

## 3. Results

Group A1, where the traditional technique was applied, showed more than 25% and 50% of debris, while Group A2, where the new technique was performed, showed no debris or less than 25%. There was a statistically significant difference between groups A1 and A2.

**Figure 2:**
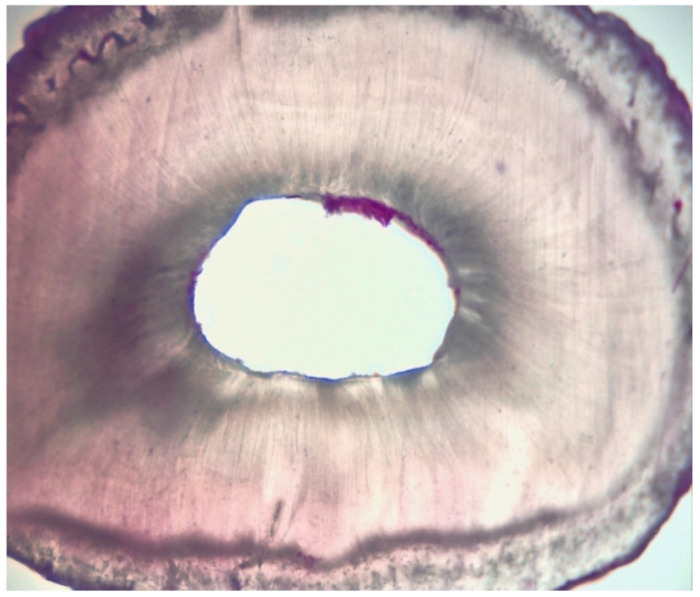
GROUP A1: Root cross-section of 6 microns thick.

**Figure 3:**
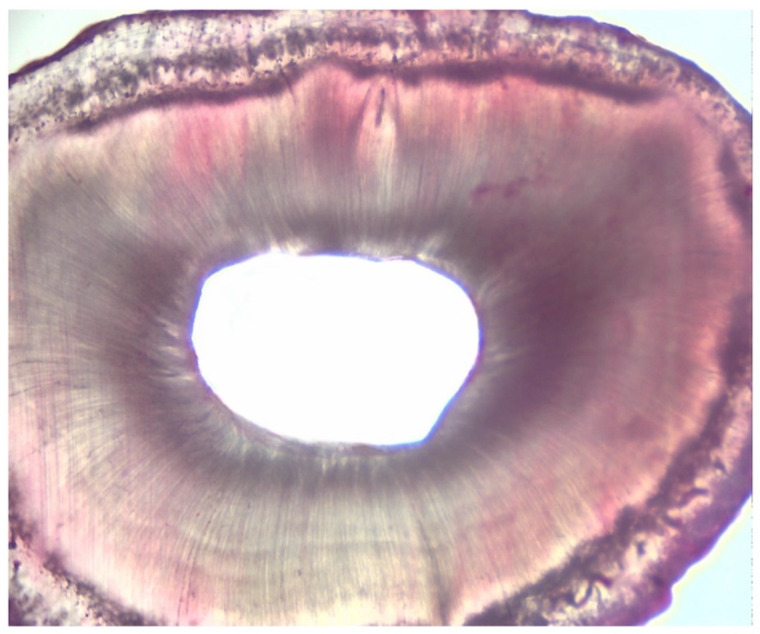
GROUP A2: Root cross-section of 6 microns thick.

Table 2, Table 3 and Table 4 summarise the results regarding tissue debris amount in the experimentation attained next to the retro-cavity preparation in groups 1 and 2. In the experiment, the amount of hard tissue debris was significantly greater in group A1 when compared to group A2 (*p* < 0.05) (Figure 2 and Figure 3).

Table 3 and Table 4 score of the degree of dissolution of the debris.

## 4. Discussion

Surgical endodontics is a clinical approach that involves cutting a portion of the apical third of the root. In particular, this part of the root is characterised by anatomical complexity, making it challenging to achieve adequate cleansing, especially in cases where it is impossible to obtain a complete seal through a non-surgical approach [15]. Moreover, the main objectives of the surgical approach are to enclose the microorganisms in the root canals by forming an apical seal, to eliminate one of the most apical and complex portions of the root canal, and finally, to eradicate any existent periapical lesions [16]. However, surgical retreatment has restricted indications, such as in cases where the canal is obstructed, and the hazard of damage to the tooth crown or restoration is extreme and possible [17].

The endodontic surgery technique had about 60% of success in the past. Such a not particularly encouraging percentage was attributable to the operational hardships in identifying, cleaning, and obturating the apical portion of the endodontic system and the use of outdated filling materials [18]. In the 1990s, the introduction of the surgical microscope [8], combined with ultrasonic retro-tips, opened the door to a new era of surgical endodontics. 

Consequently, using the operating microscope in surgical endodontics has raised success rates due to the greater precision at all procedure stages [9].

Currently, the introduction of ultrasonic retro-tips offers a more conservative and meticulous root preparation. 

The micro-ultrasonic advancements enabled dental surgeons to attain more predictable surgical outputs with increased success rates [19]. 

On the one hand, there has been a significant improvement in the quality of the cleaning of the retro-preparations. The cleaning phase of the retro cavities is a process that can still be improved to obtain a better surgical endodontic treatment success rate.

Generally, it was described that ultrasonic activation during the cleansing phases in orthograde treatment improves irrigation insertion and agitation via the physical phenomena of flow and cavitation. In vitro, the ultrasonic activation improved canal cleaning, irrigant penetration into the root canal system, soft tissue debridement, and smear layer and biofilm removal. Furthermore, the additional use of EDTA has been correlated with an added improvement in removing the smear layer due to its ability to chelate hard tissues and the de-calcifying action [2]. 

Thereupon, the current research is based on these observations to present a new cleansing protocol with activated irrigants in the retro cavities. By combining the latest technologies with the advances in irrigant activation, our proposed technique can rationally improve the success rates of surgical endodontic treatments, which, even today, are around 91% [13,20].

Previously, questions were raised regarding the amount of tooth reduction during the apicoectomy phase. There is no consensus on how much of the root must be removed to meet biological principles. Gilheany et al. [21] recommend removing at least 2 mm to reduce bacterial leakage from canals. However, Kim et al. [22] discovered that removing at least 3 mm of the root tip is required to reduce 98% of the apical ramifications and 93% of the lateral canals in an anatomical study of the root apex. The literature recommends a 3 mm root end amputation because it leaves an average of 7 to 9 mm of the root, which provides sufficient strength and stability in standard-length teeth. Briefly, a root-end amputation of less than 3 mm is likely to miss some lateral canals and apical ramifications, increasing the risk of reinfection and eventual failure. Our study compares two protocols.

The idea of the currently proposed technique is based on a conservative approach by cutting less apical parts than traditional techniques. Then, after performing a standard retro preparation with retro tip and saline solution, a small ultrasonic tip 25.02 activates EDTA and NaOCl gel to ensure deeper cleaning. Another research [23] evaluated the activation of EDTA alone and showed great results. Instead, in this work, in addition to the use of EDTA, NaOCl gel was also added. Furthermore, adding NaOCl gel aimed to decrease bacterial load deeper after removing the smear layer with EDTA.

The in vitro results of our research are promising based on the data analysis; a statistically significant difference was observed between the amount of debris present in the canals of the A1 group teeth (i.e., those irrigated with a traditional cleaning protocol) and those present in the canals of the teeth of group A2 (those irrigated with the new protocol). Furthermore, the latter has a minor amount of debris on the canal walls. The differences between the two surgical protocols can be traced back to the apicoectomy and retro-cavity cleansing phase. In particular, the apicoectomy of the new protocol involves the resection of only 1 mm of the apical third compared to the 3 mm recommended in the standard protocol. [13,15,24,25] This choice is justified by the fact that the retro cavity’s subsequent cleansing phase involves the use of EDTA and NaOCl gel and the ultrasonic activation of both agents. Consequently, with a better diffusion of the irrigants in the lateral anatomies, there is no need to have excessive tissue removal from the apical root. In addition, this proposed conservative approach is advantageous in preserving the apical part of teeth, especially with an unfavourable crown/root ratio. 

In brief, improving the quality of cleansing in the retrograde cavity may decrease the amount of cut of the apical portion of the root. This way, teeth with an unfavourable crown-to-root ratio, teeth with little periodontal support, or teeth already surgically treated with the new protocol could be treated effortlessly and effectively without further reducing the root apex.

The current study’s limitation was that the antimicrobial efficacy of the newly proposed technique needed to be tested.

## 5. Conclusions

The proposed technique showed significant differences between the groups, suggesting that using this conservative approach in endodontic surgery not only preserves the crown root ratio but can also increase the success rate by adequately cleansing the retro cavities through the use of ultrasonic-activated irrigants. In particular, the minimal cutting of apical root combined with ultrasonic activation of irrigants in retro cavities will provide a possibility to save teeth with a compromised crown-root ratio that otherwise would be doomed for extraction. 

Further research, such as an antimicrobial-based assessment method, SEMn or Micro-CT analysis, to confirm the validity of this new protocol will be employed to confirm this study’s conclusion.

## Figures and Tables

**Table 1:** Grading.

Grading	
I	presence of debris within the area
II	presence of debris in more than 50% of the entire area
III	presence of debris in more than 25% of the entire area
IV	absence of debris or presence of debris in less than 25% of the entire area

**Table 2:** Median [interquartile range] for experiment A (assessment of root end cavity cleanliness), acquired from retro-preparation in mandibular premolars in both groups 1 and 2.

	Experiment A
**Group 1**	2 [0.5] ^A^
**Group 2**	4 [1] ^B^

Different superscript letters indicate statistically significant differences between the groups (*p* < 0.05).

**Table 3:** Group A1: evaluation of the degree of dissolution of the debris.

*No.*	*A1* *1*	*A1* *2*	*A1* *3*	*A1* *4*	*A1* *5*	*A1* *6*	*A1* *7*	*A1* *8*	*A1* *9*	*A1* *10*	*A1* *11*	*A1* *12*	*A1* *13*	*A1* *14*	*A1* *15*	*A1* *16*	*A1* *17*	*A1* *18*	*A1* *19*	*A1* *20*
*Score*	I	II	II	I	II	III	II	II	II	I	II	I	II	III	II	II	III	II	I	II

**Table 4:** Group A2: evaluation of the degree of dissolution of the debris.

*No.*	*A2* *1*	*A2* *2*	*A2* *3*	*A2* *4*	*A2* *5*	*A* *2* *6*	*A2* *7*	*A2* *8*	*A2* *9*	*A2* *10*	*A2* *11*	*A2* *12*	*A2* *13*	*A2* *14*	*A2* *15*	*A2* *16*	*A2* *17*	*A2* *18*	*A2* *19*	*A2* *20*
*Score*	III	IV	IV	III	IV	III	III	IV	III	IV	IV	III	IV	IV	III	III	IV	IV	IV	IV

## Data Availability

Not applicable.
